# Observational threat learning influences costly avoidance behaviour in healthy humans

**DOI:** 10.1038/s41598-024-65602-3

**Published:** 2024-07-28

**Authors:** Madeleine Mueller, Oded Cohen, Tomer Shechner, Jan Haaker

**Affiliations:** 1 Department of Systems Neuroscience, University Medical Center Hamburg-Eppendorf (Germany), Martinistr.52, 20251 Hamburg, Germany; 2 School of Psychological Sciences and the Integrated Brain and Behavior Research Center, University of Haifa, Haifa, Israel

**Keywords:** Learning and memory, Human behaviour

## Abstract

Avoidance is an essential behaviour for ensuring safety in uncertain and dangerous environments. One way to learn what is dangerous and must be avoided is through observational threat learning. This online study explored the behavioural implications of observed threat learning, examining how participants avoided or approached a learned threat and how this affected their movement patterns. Participants (n = 89) completed an observational threat learning task, rating their fear, discomfort, and physical arousal in response to conditioned stimuli. The retrieval of learned threat was reassessed 24 h later, followed by a reminder of the observed threat associations. Participants subsequently completed a computerised avoidance task, in which they navigated from a starting point to an endpoint by selecting one of two doors, each associated with either safety or danger, relying on observed information. Opting for the safe door entailed increased effort to attain the goal. Results demonstrated that observational threat learning influenced avoidance behaviour and decision-making dependent on baseline effort level. Participants tended to exhibit thigmotaxis, staying close to walls and taking extra steps to reach their goal. This behaviour indirectly mediated the number of steps taken. This study provides valuable insights into avoidance behaviour following observational threat learning in healthy humans.

## Introduction

As social animals, humans can learn from the experiences and responses of others. Consequently, threat learning does not occur solely through firsthand experience; it can also be achieved through observation in a process known as social or observational threat learning^1^. This type of threat learning allows individuals to benefit from the mistakes or risky behaviour of others by interpreting (conspecific) threat expressions and thereby avoiding dangerous consequences for themselves.

In a laboratory setting, observational threat learning can be employed when participants watch a video of a demonstrator undergoing a classical threat conditioning paradigm^2^. In this video, the demonstrator encounters two types of stimuli. The first type, known as the observed conditioned stimulus (OCS +), is followed by an aversive outcome for the demonstrator, termed the observed unconditioned stimulus (OUS). This pairing establishes the OCS + as predictive of threat. Conversely, the second type of stimulus (OCS-) is not followed by the OUS and therefore does not predict threat. The term 'OCS' refers to the observational stimuli (videos of the demonstrator undergoing aversive learning). Subsequently, participants are directly confronted with the stimuli that were learned as dangerous or safe by responses of the demonstrator, i.e. the 'CS'. The observing participant never experiences an aversive outcome firsthand; but instead they learn the contingency between the OCS + and OUS by observing the demonstrators’ responses^3^.

In order to respond adaptively to threats in the environment, it is key that learning is transferred into behaviour. In many species, including humans, it has been shown that information learned through observation can be transferred into new behaviour. This includes the use of tools^4^, vocal imitating^5^ or transfer of observational threat learning to instrumental behaviour^2,3,6^. Once a threat has been detected, one effective strategy is to avoid confrontation with the potentially dangerous outcome ^7^. Avoidance of learned fear is defined as the prevention of the occurrence of a threat-related stimulus (i.e. conditioned stimuli (CS) avoidance)^8^ and has been found following observational threat learning in rodents^6^ and toddlers^9^. Avoiding a threat-related stimulus might reduce the anticipated danger, but it can also be costly. For instance, spotting a snake on the road in the distance could lead an individual to either proceed down that road, potentially risking a snakebite, or opt for an alternative route that avoids confrontation with the threat, which might be safer but longer (i.e., more costly). In everyday life, avoidance is only an adaptive strategy when it is carefully balanced against costs and gains of safety. Classical behavioural avoidance protocols frequently implemented a threat learning phase, followed by an avoidance task^10^. Here, we implemented an observational threat learning task that was followed by a spatial navigation task that included a decision to avoid or approach the CS + in a freely chosen route.

In our computerised experiment we thought to realise the balance between costs and safety as the increased number of steps that participants had to take to avoid the CS + (i.e., costly avoidance). To this end, participants could choose their routes freely from a start-point to a designated goal-point by deciding to cross a field that contained either the CS + or the CS-. A shorter route was possible when participants decided to cross the socially learned threat (CS +), while a longer (more costly) route allowed to avoid the threat (i.e., to crossing the CS-), but required more steps to reach the goal. Previous studies employing costly avoidance designs have demonstrated that reduced avoidance costs result in more pronounced avoidance behaviour^11^. The aim of our study was to investigate whether threat learned by observation affects costly avoidance behaviour and decision-making in healthy humans. There is evidence that human participants learn to avoid threats by social observation^12^, but investigations of decision making underlying costly avoidance of socially learned threats is scarce. By implementing a costly approach-avoidance task that permitted participants to move freely before and after decision-making, we allowed them to adapt their behaviour depending on the required effort. We expected participants would choose a longer and more effortful path to avoid threats that are learned by observation. This would indicate a behavioural transfer in the form of active avoidance.

## Results

### Observational learning

**Figure 1:**
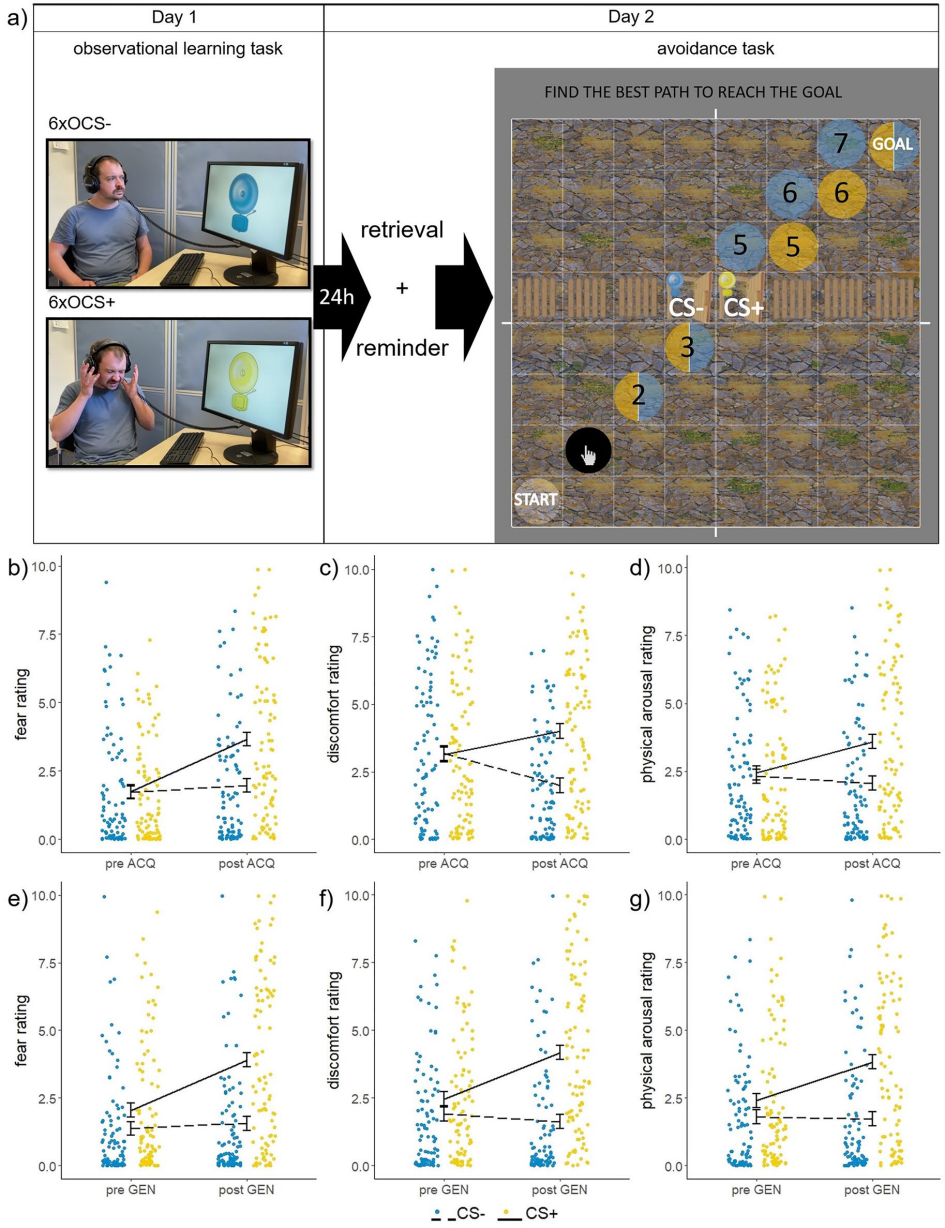
(**a**) Paradigm. The CS + (yellow) and the CS − (blue) were learned during the observational learning task on day 1. In the avoidance task on day 2, the white circle is the current position of the participant. The black circle indicates a possible next movement that was highlighted when hovered over with the mouse. Exemplary version left → right/short pathlength. Optimal routes are indicated by colour-coded circles (CS + steps = 7, CS- steps = 8). Colours were counterbalanced. (**b**–**g**) Rating results before and after observational learning task (**b**–**d**) and generalisation task (**e**–**g**). Participants learned to discriminate between stimuli during observational learning (ACQ), showed a retrieval of learned discomfort and physical arousal on day 2 and increased their stimulus discrimination again during day 2 following the reminders during generalisation (GEN) in all three ratings.

As expected, we found a stimulus*time interaction in all three ratings, indicating successful observational threat learning (Supplementary Table S1). No differences between the CSs were found in all three ratings before the experiment started. However, following the observational learning task, participants rated their fear, discomfort and physical arousal higher when presented with the CS + , than the CS − (see Fig. 1b–g and Supplementary Table S1). The discrimination (CS +—CS −) post learning was larger than before learning (time main effect: discomfort: F(1,89) = 36.463, *p* < 0.001, post–pre: t(89) =  − 6.038, *p*_corr_ < 0.001; fear: F(1,89) = 37.48, *p* < 0.001, post–pre: t(89) =  − 6.123, *p*_corr_ < 0.001; physical arousal: F(1,89) = 27.245, *p*_corr_ < 0.001, post–pre: t(89) =  − 5.220, *p* < 0.001).

### Behavioural transfer/avoidance task

**Figure 2:**
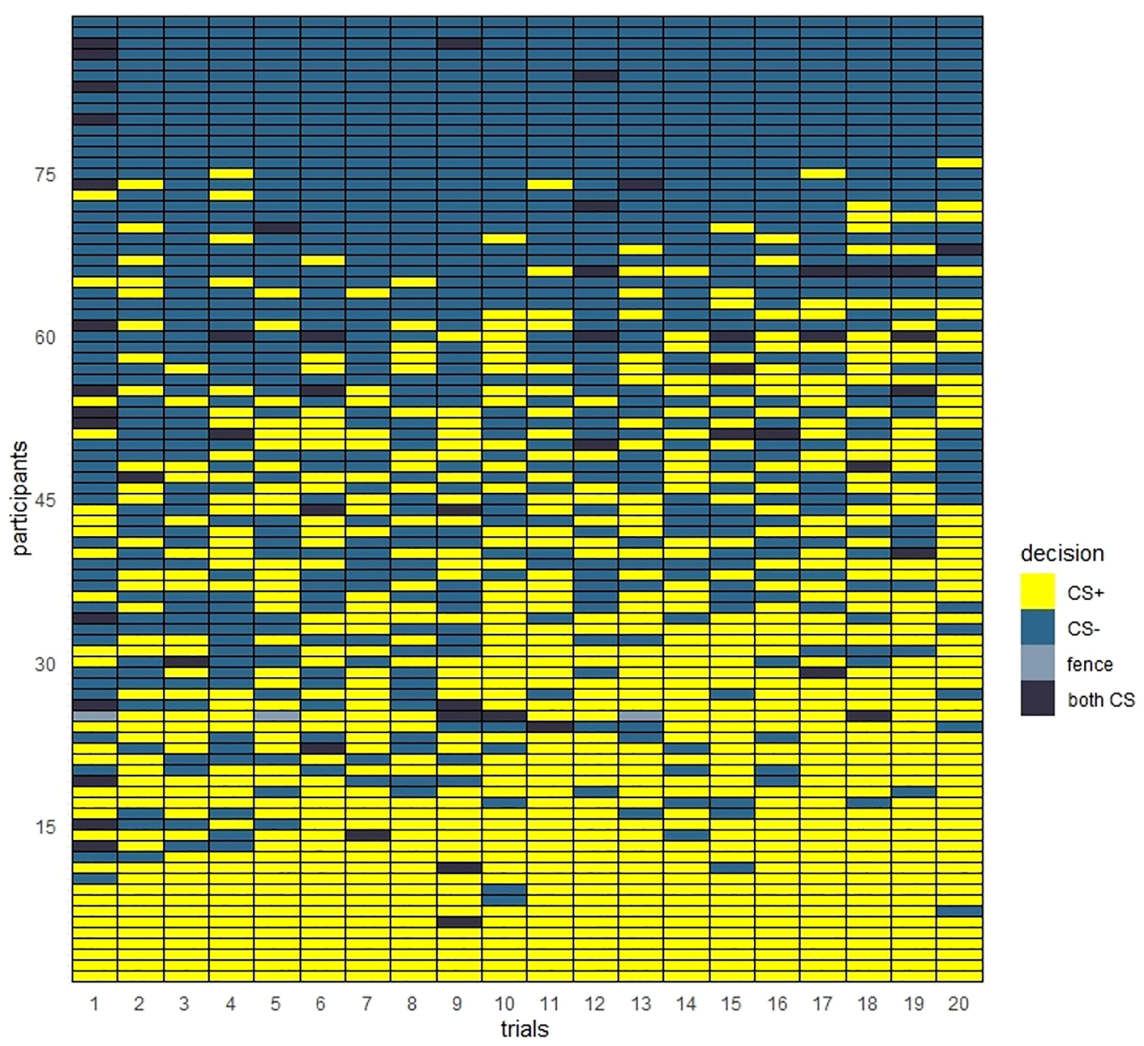
Door decisions in all 20 trials ordered by the total number of CS + door decisions for each participant from highest (bottom) to lowest (top).

A descriptive analysis of CS + and CS − door decisions in each of the 20 trials per participant revealed that avoidance behaviour was heterogenous across participants (see Fig. 2). Six participants never used the CS − door (never avoided the CS +), whereas 13 participants never used the CS + door (always avoided the CS +). Descriptively, the majority of participants used the CS − door in the first trial, which they had learnt was safe (51.7% of the participants used the CS − door, 31.5% used the CS + door, 1.1% used the fence and 15.7% tried both doors in the first trial).

### Step analysis

**Figure 3:**
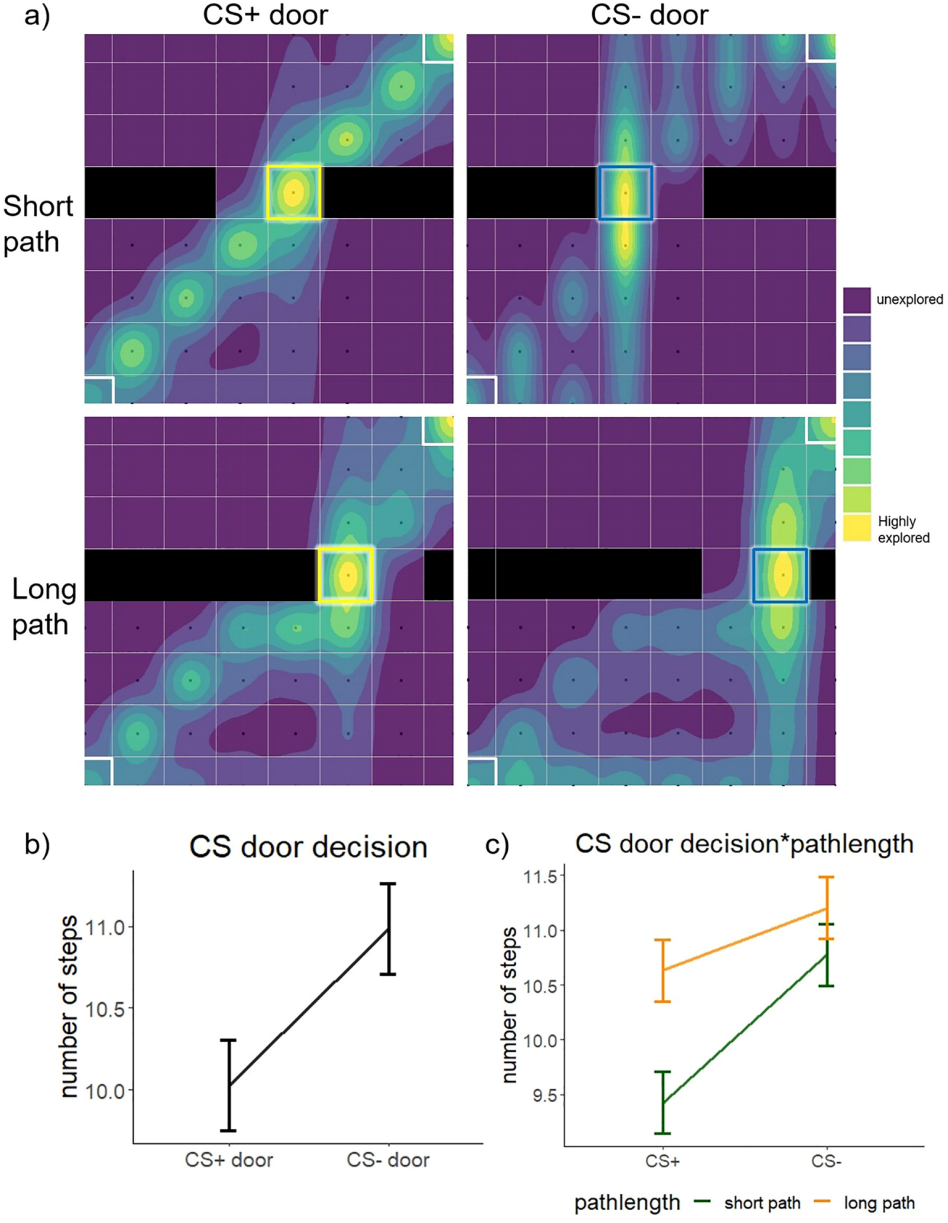
(**a**) Heatmaps of movement through grid depending on pathlength and CS door decision. The yellow square indicates the position of the CS + door, the blue square indicates the position of the CS − door and the white square indicates the position of the start/goal. Path directions from bottom right to top left were mirrored on the vertical axis and are included in the figure. Violet colours indicate a low number of times participants stepped on this tile, and yellow colours indicate a high number of times participants stepped on this tile. Black dots indicate at least one step on that specific tile. (**b**) Participants needed more steps when walking through the CS − door, (**c**) and this was dependent on the pathlength.

As predefined by the paradigm, we found a main effect on the number of steps based on the CS door decision (F(1,1635.06) = 118.632, *p* < 0.001), with a higher number of steps taken when choosing the CS − door compared to the CS + door (t(1645) = 10.121, *p*_corr_ < 0.001) (see Fig. 3b and Supplementary Table S2). This suggests that participants invested greater effort (i.e., took more steps) to avoid the CS + door, which aligns with the experimental design. Importantly, we also found a CS door decision*pathlength interaction (F(1,1618.77) = 16.672, *p* < 0.001). This interaction suggests that under conditions of generally lower effort (short path condition), participants were more inclined to invest additional effort to avoid the CS + door. In contrast, under conditions of generally higher effort (long path condition), the additional number of steps to avoid the CS + door was lower, compared to the short path (CS + door_mean steps_ – CS − door_mean steps_ per pathlength, i.e. the lower the mean, the more steps were taken during the CS- decision trial: main effect pathlength: F(1,58.57) = 11.411, *p* = 0.001; short pathlength – long pathlength: t(58.6) =  − 3.378, *p*_corr_ = 0.001; see Fig. 3c).

Because the difference between the location of the CS + door and the location of the CS − door was always one step (in both the long and short path conditions), this interaction further suggests that the difference in number of steps between the CS + and CS − does not solely reflect the task demands.

Furthermore, we found a main effect of trial (F(19,1608.20) = 16.672, *p* < 0.001). As depicted in Supplementary Figure S2, the number of steps per trial is decreased over time. One possible explanation is that the effort required for clicking exceeded the desire to explore the grid over time (trial 1 – trial 20: t(1609) = 11.072, *p* < 0.001), and this was coupled with the extinction of the CS + following repeated firsthand exposures to the CS + without aversive outcomes.

### Rating + decision analysis

As a next step, we tested if stimulus ratings before starting the avoidance task on day 2 and the CS door decision were linked. We found a main effect of discomfort ratings (F(1,1714) = 23.772, *p* < 0.001; see Supplementary Figure S4a) on the CS-door decision. Participants who rated the CS + as more discomforting than the CS − on day 2 showed more avoidance behaviour of the CS + door. Furthermore, we found a main effect of pathlength, indicating participants decided more often to use the CS + door when confronted with the long path (F(1,1714) = 9.347, p = 0.002; short path – long path: z = 3.063, *p* = 0.002; see Supplementary Figure S4b). As the general effort level was higher in the long path, this seems to have led the participants to choose the CS + door, as it needed fewer steps compared to the CS- door. We found no main effects of differential fear or physical arousal ratings on day 2 (fear: F(1,1714) = 1.980, *p* = 0.160; physical arousal: F(1,1714) = 0.468, *p* = 0.494).

### Reaction time analysis

**Figure 4:**
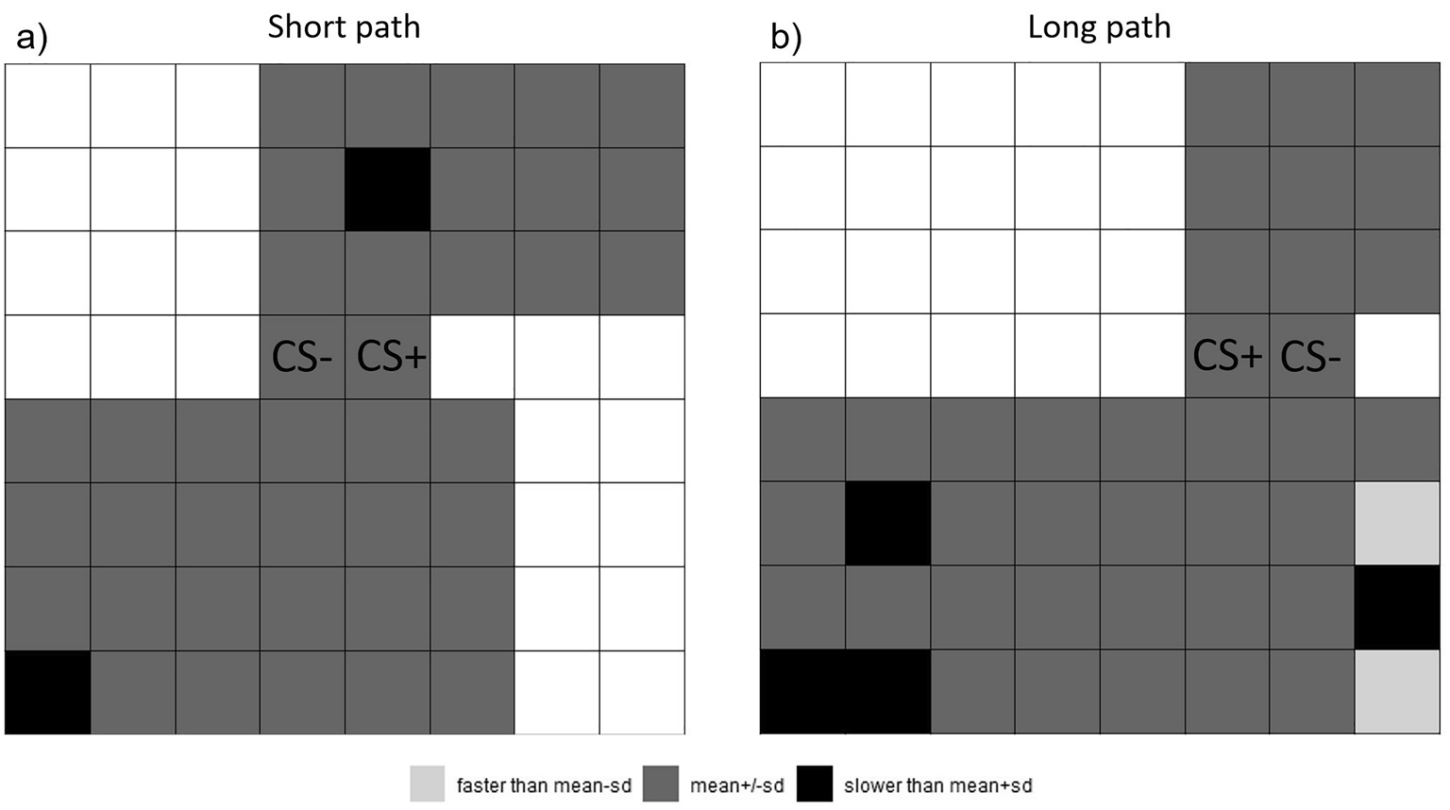
Mean z-transformed reaction time over all tiles visualised for each pathlength. Colour indicates if the mean z-transformed reaction time was faster or slower than the mean + / − standard deviation.

To determine whether the reaction times differed for tiles in different parts of the grid, we calculated the total mean z-transformed reaction time with standard deviation. Next, we compared the mean z-transformed reaction time of each tile with the total mean z-transformed reaction time. Figure 4 illustrates the results separately for both pathlengths. Participants spent longer than the mean reaction time on the very first tile in both pathlengths. This result might indicate a first orientation at the beginning of the route. There were three additional tiles in the long path where participants spent longer time than the mean reaction time before the CS door decision, all of which were directly on or in close proximity to the edge of the grid. This could indicate a longer decision time before deciding on a specific CS door. Furthermore, reaction speed may be enhanced in the centre of the grid, relative to the edges.

#### Pre decision

To investigate the reaction time per step before the door decision was made, we included the reaction time as the outcome measure in the linear mixed model: lmer(prereactiontime ~ (1|participant) + CS*pathlength + steps + trial (Supplementary Table S4). A main effect of trial revealed participants increased their reaction time over time (trial 1 – trial 20: z-ratio = 17.07, *p* < 0.001). Additionally, we found a main effect of steps, indicating that the more steps participants took before the decision, the faster their reaction time per step.

#### Post decision

To investigate the reaction time per step after the door decision was made, we included the reaction time as an outcome measure in the linear mixed model: lmer(postreactiontime ~ (1|participant) + CS*pathlength + steps + trial (Supplementary Table S4). Similar to the pre-decision reaction time analysis, we found a main effect of steps (F(19,7347.5) = 20.9536, *p* < 0.001) (the more steps the faster) and a main effect of trial (F(1,7237.9) = 168.1539, *p* < 0.001) (the later the faster).

### Wallfactor analysis

**Figure 5:**
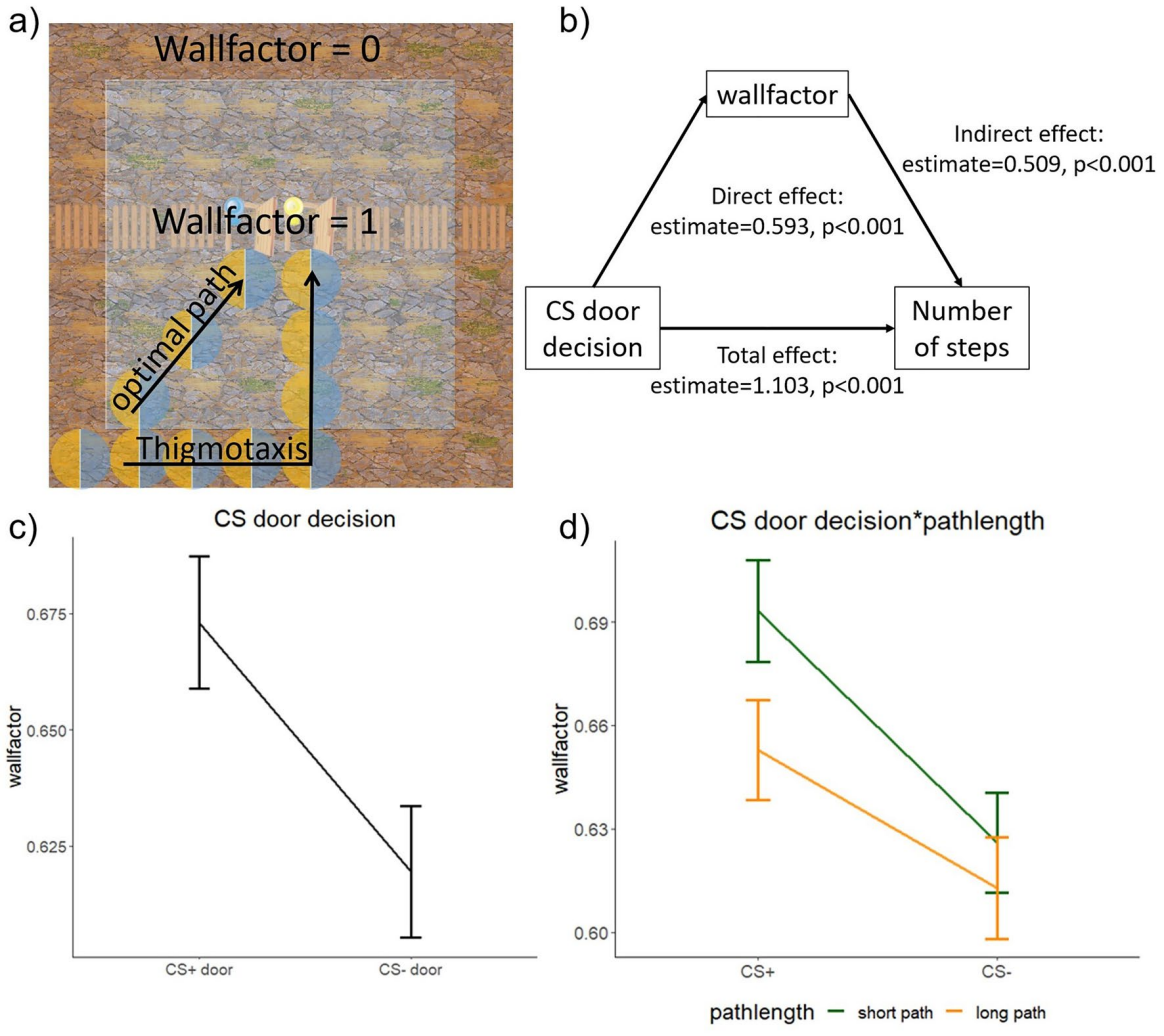
(**a**) The wallfactor of the steps taken on the tiles directly at the wall was 0. The wallfactor of the steps that were taken on the tiles in the centregrid was 1. This was included into the analysis to investigate the thigmotaxis behaviour of the participants. (**b**) A partial mediation was found between the CS door decision and the number of steps, mediated by the wallfactor. (**c**) A higher wallfactor indicates more movement through the centre of the grid; wallfactor = 0, whenever participants moved directly at the wall, and wallfactor = 1 if participants moved through the remaining centre of the grid. Participants moved closer to the wall when they chose the CS − door then when they chose the CS + . (**d**) This effect was stronger in the short path.

The movement heatmaps (see Fig. 3a) revealed that some participants had a tendency to move closer to the wall, rather than taking the shortest path through the centre of the grid. This behaviour resembles thigmotaxis, the behaviour to stay closer to walls, rather than moving in open spaces (see Fig. 5) ^13^. To investigate the role of this behaviour, we defined a wallfactor variable: 0 whenever participants moved directly by the wall and = 1 if they moved through the remaining centre of the grid. That means the smaller the wallfactor, the more participants moved along the wall and avoided the centre grid (Fig. 5a). We included the wallfactor as an outcome measure in the model: lmer(wallfactor ~ (1|participant) + CS*pathlength + trial (see Fig. 5 and Supplementary Table S5) and found a CS by pathlength interaction (F(1,1630.46) = 7.140, *p* = 0.008, see Fig. 5d). Additionally, we found a main effect of CS (F(1, 1654.93) = 70.1281, *p* < 0.001). Post-hoc tests revealed that when a participant decided to use the CS- door, they avoided the centre grid, compared to when they chose the CS + door (CS +—CS-: t(1668) = 8.732, *p* < 0.001). Hence, avoidance of the learned threat (i.e., CS +) was related to avoidance of the centre (i.e., thigmotaxis). Furthermore, a main effect of pathlength (F(1,1623.36) = 33.4875, *p* < 0.001) showed that participants avoided the centre grid more strongly in the long path (short path – long path: t(1614) = 5.724, *p* < 0.001). We also found a main effect of block (F(19,1613.3) = 5.4230, *p* < 0.001). A post-hoc test comparing the first and the last trial revealed that the wallfactor increased over time (trial 1 – trial 20: t(1614) = -4.909, *p* < 0.001) meaning participants tended to walk through the centre in later trials. This can be explained by taking shorter paths/fewer steps over time to minimise effort, but also by the extinction of the threat.

### Mediation analysis steps—wallfactor—CS door decision

In our analysis, we found that both the CS door decision and thigmotaxis had an impact on the number of steps in each trial. To investigate if thigmotaxis (= lower wallfactor) might mediate the effect of the CS door decision on the steps taken, we conducted a model-based mediation analysis using the mediation package in R (mediator model: lmer(wallfactor ~ (1|participant) + CS); outcome model: lmer(steps ~ (1|participant) + CS + wallfactor); simulations = 1000).

The results revealed an effect of the CS door decision on the number of steps (estimate = 1.103, 95% CI [0.898, 1.31], *p* < 0.001). When wallfactor was added to the association between the CS door decision and steps, there was a direct effect of the wallfactor onto steps (estimate = 0.593, 95% CI [0.407, 0.79], *p* < 0.001). In addition, there was an indirect effect (estimate = 0.509, 95% CI [0.392, 0.63], *p* < 0.001), indicating a partial mediation between the CS door decision on the number of steps via wallfactor (see Fig. 5b).

### Rating results connected to step and decision based analysis

In order to test whether ratings of discomfort, fear, and physical arousal towards the CSs were associated with the number of steps (i.e., effort) participants took when opting for either the CS + or the CS − , the following model was applied: lmer(steps ~ (1|subject) + ratingdifference*CS*pathlength + trial).

**Figure 6:**
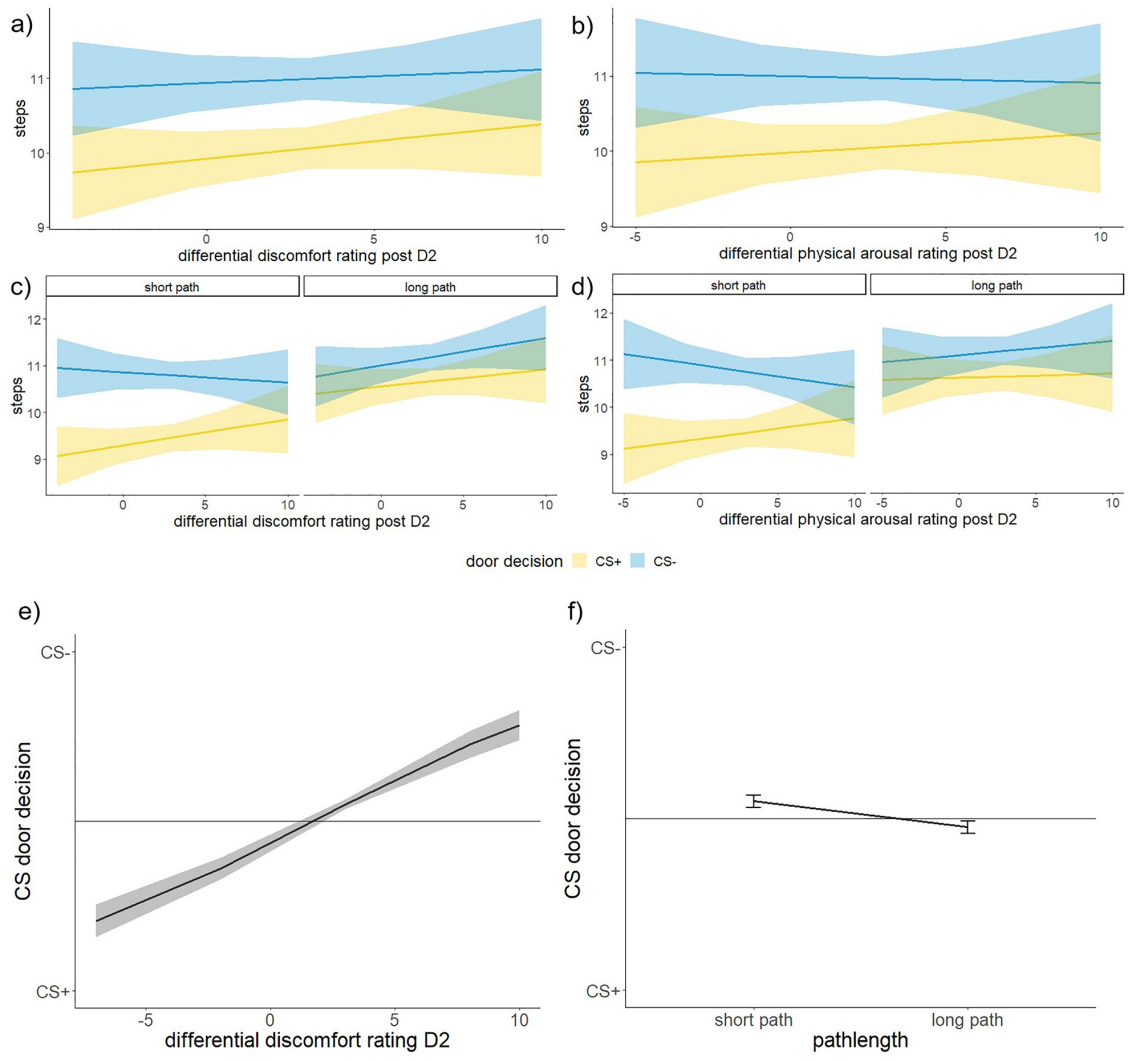
(**a**–**b**) Stimulus discrimination in the discomfort and physical arousal ratings directly before starting the behavioural transfer and the steps that were taken are related. (**c**–**d**) This relation is most pronounced in short pathlength. (**e**) Stimulus discrimination in the discomfort ratings before starting the behavioural transfer and the CS door decision are related. The stronger the discrimination in the discomfort rating (CS +  − CS − , post–pre) the more likely the CS − door was to be chosen. (**f**) When confronted with an increased general effort level (long pathlength), participants more often decided to choose the CS + door than the CS − door. This might be explained by the attempt to decrease the required number of steps.

Stimulus discrimination in the ratings directly before starting the avoidance task (‘post GEN’) and the number of steps were linked. We found a differential rating*CS interaction in the discomfort and physical arousal ratings (discomfort: F(1,1628.70) = 4.0176, *p* = 0.04519; physical arousal: F(1,1630.88) = 4.3780, *p* = 0.03656), but post-hoc analyses found no difference in slopes between CSs (CS + – CS − ; discomfort: t(1637) = 0.954, *p* = 0.34, physical arousal: t(1640) = 1.042, p = 0.298). Furthermore, we found a differential rating*CS*pathlength interaction in the discomfort and physical arousal ratings (discomfort: F(1,1615.89) = 3.9566, *p* = 0.04686; physical arousal: F(1,1615.63) = 4.0594, *p* = 0.04409) (Fig. 6a–d and Supplementary Table S3). Here, we found a difference in slope between CSs in the short path condition for both, discomfort and physical arousal ratings (CS + – CS − ; discomfort: t(1629) = 2.00, *p* = 0.045, physical arousal: t(1631) = 2.092, *p* = 0.037), but not in the long path condition (CS + – CS − ; discomfort: t(1628) =  − 0.57, *p* = 0.569, physical arousal: t(1630) =  − 0.486, *p* = 0.627).

The interaction between ratings and CS indicated that the number of steps was associated with the learning success and the affective evaluation of the threat and safety signals, respectively (Fig. 6a–b). The additional interaction with pathlength further suggests that this association (number of steps with threat learning) was not found when participants chose the CS − door in the short path (see Supplementary Figure S3c–d). In this condition, an additional factor may have contributed to the number of steps taken (see mediation analysis).

### STAI-T and STAI-S questionnaires

Including the results of either the trait anxiety questionnaire (STAI-T) or the state anxiety questionnaire (STAI-S, prior to day 1 or day 2) showed no results in the ANOVA.

## Discussion

Our main findings demonstrate that threat learning by social observation shapes avoidance behaviour in the observer. We found that participants avoided integrating threat-related objects (i.e., CS +) into a route, even when this avoidance required more effort in terms of a longer route. Previous studies have shown that avoidance learning via instructions and social observation can be just as successful as direct learning^12^, and observational threat learning leads to avoidance of the threat in the observer^9^. Here, we demonstrate that observational learning impacted the balance between costs and avoidance behaviour in the observer. As such, avoidance behaviour was dependent on the baseline effort predetermined by the paradigm. The more effort required from the start (i.e., long pathlength), the lesser the difference in effort (i.e., steps) between the decision to take the route through the CS + or the CS − . It is likely that participants perceived the longer route to be more demanding in general, leading them to attempt to reach their destination with less deviation from the optimal path.

Furthermore, the individual ratings of discomfort elicited by the observation-learned stimuli were associated with the CS door decision. With higher ratings of discomfort (difference between the CS + and CS −), participants were more inclined to choose the CS − door, indicating a preference for the safer, less discomforting option, despite additional effort.

Interestingly, we found another factor was associated with more effort (i.e., more steps) in navigating through the grid, namely a preference to avoid the centre. When seeking safety from (aerial) predators, animals such as rodents tend to stay close to walls, rather than exploring the open centre of a field^14^. A paradigm to investigate this avoidance behaviour is the open field test, in which centre avoidance in a novel open space is measured. This behaviour, known as thigmotaxis, has also been found in humans^15,16^. Increased thigmotaxis can prolong the assessment of novel environments. This prolonged activity is linked to diminished working memory and a decreased ability to create an overall spatial perception in healthy humans^17^. A study in patients suffering from agoraphobia and healthy participants with high anxiety sensitivity found increased thigmotaxis, when compared to healthy controls and low anxiety sensitive participants^15^. In our study, we found that participants took extra steps to reach the goal. While one contributing factor to the number of steps was avoidance of the CS + , the movement patterns suggest that participants preferred to stay close to the wall and only left it when walking through the door. This behaviour can be explained by thigmotaxis as participants tried to stay closer to the wall, as this context was new and potentially threatening. Moreover, the preference to stay closer to the wall (i.e., wallfactor) was an indirect mediator of the number of steps when avoiding the CS + . Hence, the number of steps was increased when participants avoided a socially learned threat (CS +), mediated by the preference to stay closer to the wall, suggesting a typical movement pattern of anxiety related behaviour.

Such an interpretation is further supported by the analysis of the response times. In our experiment, participants could navigate the grid freely, not just spatially but also temporally. This setup created a more natural environment, allowing participants to determine for themselves when they were prepared to make a decision. Notably, response times were longest at the start of the grid and within positions that were closer to the walls, suggesting participants did not immediately initiate movement, but took time to assess the situation before navigating, which is also suggested by our interpretation of the thigmotaxic behaviour. Pre-decision response times were potentially influenced by processes such as information gathering, risk–benefit assessment and strategic planning and these may have led participants to move away from their mean pre-decision speed, especially in the long path version.

The number of steps diminished in later trials, possibly because of reduced motivation to deviate from the optimal path. Choosing a non-optimal path necessitated greater effort in clicking and extended experiment completion times. Another plausible explanation is that participants learned over time that neither CS door was linked to adverse outcomes (extinction), thus reducing the number of steps taken. A third possibility is that growing familiarity with the context over time led to reduced thigmotaxis, resulting in fewer required steps. Of course, it is likely that all three options explain the final behaviour.

A limitation of this study was the reduced control over participants' adherence to the study protocol, due to its online nature. It is also important to mention that the effort of the virtual movement patterns cannot be transferred one-to-one to a standard open field test or to efforts to move within real environments. In contrast, our paradigm demanded a predefined a goal-directed movement that forced the participants to move through the centre of a virtual room and decide between two associatively learned stimuli. Another limitation is that our study design included only two different levels of effort (short vs. long path). For a more diverse view of effort-based decision-making, it might be helpful to include more levels of effort, for example, a greater difference between danger and safety options. It would also be interesting to include self-reports of participants' perceptions of effort. Another option would be to have the same level of effort for both CSs. In addition, measurement of explicit US expectancy and physiological responses during the UCR would provide insights into other aspects of observational learning. Finally, we did not find an effect of trait or state anxiety on avoidance behaviour, which might be due to a more complex influence of anxiety on our task (e.g. mediation or moderation), or it may be a specific limitation of our sample (e.g., number or participants or variation in the measurement).

In conclusion, our results provide evidence that observational threat learning shapes decision-making processes and movement patterns in the context of safety and danger associations.

## Material and methods

### Stimulus materials

Pictures of either yellow or blue doorbells served as CSs (based on ^18^). Colours of the CS + and CS − were counterbalanced. In the following, we will refer to CSs as to the stimuli that were directly presented to participants. We refer to the observational stimuli (video) as ‘OCS’. An observational unconditioned stimulus is referred to as the OUS. OCSs were presented in 12s videos in which a male demonstrator wearing headphones was looking at a monitor displaying either the CS + or the CS − . The onset of the OUS was marked by the demonstrator's facial reaction to an unpleasant sound, occurring 7 s into the video and lasted 1 s.

Generalised stimuli were five doorbells with a colour gradient ranging from yellow and to blue (based on ^18^).

### Experimental procedure

#### Observational learning

Participants took part in the study online by filling out the questionnaires (State-trait-anxiety-inventory (STAI-S/STAI-T), completed with www.soscisurvey.de) on day one. This was followed by the *observational learning task* (completed with PsychoPy3). Here, participants observed a demonstrator completing two blocks of threat learning with six OCS presentations per block (3xOCS − ; 2xOCS + reinforced; 1xOCS + not reinforced; Fig. 1a). This resulted in 12 trials in total (6xOCS + , 6xOCS −) with a reinforcement rate of 66%. Stimulus order was predefined for the two blocks, but the order of the blocks was randomised. After every OCS an inter trial interval (ITI) was presented (randomised duration between 4 and 6s). Before and after the learning phase participants were asked to rate their discomfort, fear and physical arousal towards the CS + and CS − (visual analogue scale: How much discomfort do you feel confronted with this picture [CS]? 0 (no discomfort) − 10 (much discomfort); similarly for fear and physical arousal rating).

On day 2 (24h (± 6h) after day 1), participants filled out the STAI-S again, followed by a direct *generalisation task*. The results of the generalisation task are not presented here, as they are integral to a separate project conducted within a different sample. Before the generalisation task, participants were instructed to put on headphones and informed that they might hear an unpleasant sound (US). They were asked to adjust the volume on their computers to the maximum setting, and a test sound (‘beep’) was played. If they were unable to hear the sound, they were instructed to check their headphones and volume settings and attempt the test again until they could hear the sound. During the following task, no aversive sound was actually presented. The generalisation task consisted of three blocks in which five generalisation stimuli (GSs), and the two learned CSs were presented pseudorandomly. Each of the seven stimuli was presented three times, for a total of 21 trials. Following each CS and GS, an ITI was presented (randomised duration between 4 and 6s). Before the second and third block, two videos showing the reinforced OCS + and the OCS − were presented as a reminder (see Supplementary Figure S1). During each presentation of the CS/GS, participants were asked how safe/dangerous they estimated their situation when confronted with the respective stimulus on a scale of 1–4 (1 = safe/4 = dangerous). Before and after the generalization, participants were again asked for their discomfort, fear and physical arousal.

#### Behavioural transfer/avoidance task

Upon completing the generalisation task on day 2, participants proceeded with the behavioural transfer/avoidance task, while retaining the headphones from the previous task. The task was presented in an 8 × 8 tile grid. Participants started each trial from one of the bottom corners with the objective of reaching a tile labelled ‘goal’ in the opposite top corner. Each step required a click on an adjacent tile, indicated by a black circle. In row 5 of the grid, a fence barrier obstructed the path with only two doors for passage. One of the two doors had the CS + doorbell, and the other had the CS − doorbell. Participants had the freedom to choose which door to pass through in their quest to reach the goal. When participants clicked on a tile with a fence, a warning sign appeared, reading ‘stop climbing over the fences’. The instruction was to select ‘the best path’ through the grid navigating from the starting point to the goal without choosing tiles with fences. Importantly, the effort to walk through the CS − door was greater, as participants had to take at least one additional step (requiring one more mouse click) for this route than for the route that included the CS + door. Two different path directions were used (start bottom left → goal top right and start bottom right → goal top left) in two different pathlengths (the short pathlength included the shortest path through the CS + door with 7 steps and the long pathlength included 8 steps). Participants performed each of these four versions (left > right/ short pathlength; left > right/ long pathlength; right > left/ short pathlength; right > left/ long pathlength) pseudorandomly five times (20 trials).

To monitor the participant's movement within the grid, we recorded the coordinates of each position. This allowed us to keep a record of the number of steps participants took to reach the goal and which door they selected for passage. We also logged the reaction time for each step.

### Participants

**Figure 7:**
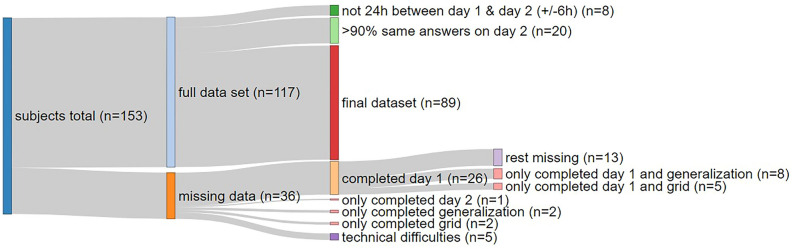
From an initial 153 participants 89 participants met all criteria to be included in the final sample (in red).

We recruited healthy individuals aged 18 to 65 years for this online study until we reached our pre-registered sample size of > 89 participants in observational learning (one additional participant was excluded from the avoidance task due to technical difficulties) ^19^. Participation necessitated the use of a computer equipped with headphones. Participants were excluded from the analysis if they did not complete all three parts of the study (observational learning, direct generalisation, and behavioural transfer/avoidance tasks) or if there was fewer than 18 h or more than 30 h (i.e., 24h ± 6 h) between the execution of observational learning (ACQ on day 1) and generalisation (GEN on day 2) tasks. Additionally, all participants who provided more than 90% uniform ratings across all same stimulus types during the safe/danger ratings on day 2 (i.e., consistently clicking through) were excluded from the analysis. Initially, 153 participants were recruited. After checking for the exclusion criteria, our final sample size was 89 participants (65 female, 23 male, 1 diverse) (see Fig. 7). Participation was compensated with 10 €. Participants answered demographic questions (age (mean age = 27.73 years, sd = 5.584, min = 19 years, max = 53 years)), gender, alcohol consumption (mean alcohol consumption = 1.053 units per week, sd = 1.583, min = 0/week, max = 8/week), coffee consumption (mean coffee consumption = 1.25 units per day, sd = 1.09, min = 0/day, max = 5/day) and smoking status (78 non-smoker, 11 smoker). They also completed the Trait-Anxiety-Inventory (STAI-T), the State-Anxiety-Inventory (STAI-S)^20^ and a questionnaire about their physical activity. Participants who smoked answered the Fagerström questionnaire for nicotine dependence^21^. The study was approved by the local ethics committee (Ethik-Kommission der Aerztekammer Hamburg, Germany, Kennzeichen: PV 5462) before the experiment was performed. All experiments were performed in accordance with institutional ethical guidelines and the Declaration of Helsinki. Informed consent was obtained from all participants, as well as from our demonstrator model, who also consented to publication of the image in an online open access publication.

### Data analysis

As preregistered^19^, we used a linear mixed model in R implemented in the lme4 package and a follow-up ANOVA Type 3 (car package). Estimated marginal means (EMMs) were then computed as post-hoc tests (emmeans package) and Bonferroni-Holm corrected.

#### Observational learning

To analyse the discomfort, fear and physical arousal ratings during the observational learning task, we used linear mixed models that included the respective ratings as the dependent variable, a random intercept for each participant, and stimulus type and timepoint as fixed effects (rating ~ (1|participant) + stimulus*time). Fear, discomfort and physical arousal ratings had two levels of stimulus type levels (CS + /CS −) and two levels of time (pre/ post). To analyse stimulus discrimination (CS + –CS −) as preregistered^19^ an additional model was created with CS differences as outcome measures (CSdifference ~ (1|participant) + time).

#### Behavioural transfer/avoidance task

The analysis of the avoidance task focused on the number of steps taken by the participants. In the linear mixed models, we included the number of steps per trial per participant as the dependent variable and added random intercepts for each participant. The CS door decision (2 levels: CS + door/CS − door), the trial-number (20 levels: 1–20) and the pathlength (2 levels: long path/short path) were entered into the model as fixed effects (steps ~ (1|participant) + CSdoor*pathlength + trial). Trials in which participants chose a path over the fence or clicked on both CS doors were excluded from analysis. Additionally, if a single step in one trial had a reaction time > 30s, this trial was excluded from the analysis.

To infer the effect of CS-specific ratings of discomfort, fear or physical arousal on the CS door decision, we used a general linear model. Here, the CS-specific ratings were computed as the differences in ratings ((CS +  − CS-)_post_ − (CS +  − CS −)_pre_) reported before the behavioural transfer/avoidance task on day 2 (glm(CS ~ discomfort + fear + physical arousal + pathlength).

### Supplementary Information

Supplementary Information.

## Data Availability

All data and code are available under this link: 10.12751/g-node.6blt1i
