# Supplementary material for: Observational threat learning influences costly avoidance behaviour in healthy humans
Source: Sci Rep. 2024 Jul 28;14:17346. doi: 10.1038/s41598-024-65602-3 (PMC11284217; doi:10.1038/s41598-024-65602-3)
Supplement: Supplementary file 1 — Supplementary Information. [file 41598_2024_65602_MOESM1_ESM.docx]

SUPPLEMENTARY MATERIAL

-

OBSERVATIONAL THREAT LEARNING INFLUENCES COSTLY AVOIDANCE BEHAVIOUR IN HEALTHY HUMANS

Madeleine Mueller^1^, Oded Cohen^2^, Tomer Shechner^2^ & Jan Haaker^1,*^

[1] University Medical Center Hamburg-Eppendorf (Germany), Department of Systems Neuroscience

[2] School of Psychological Sciences and the Integrated Brain and Behavior Research Center, University of Haifa, Israel

*Corresponding author: Jan Haaker; Martinistr.52, 20251 Hamburg, Germany; j.haaker@uke.de; phone: +49 (0) 40 7410 – 27300; Fax: +49 (0) 40 7410-59955

**Material & Methods**


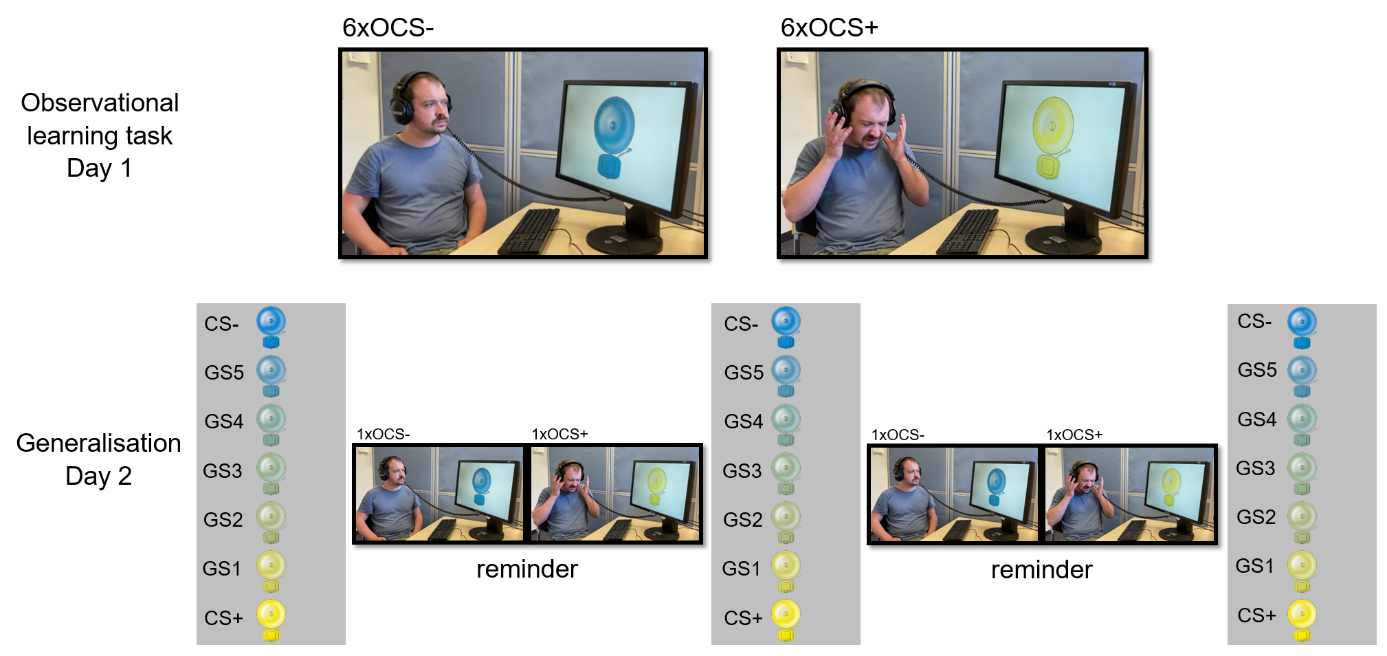


Supplementary Figure S1: Experimental procedure observational learning task and generalisation task.

**Results**

Supplementary Table S1: ANOVA results of fear, discomfort and physical arousal rating in observational learning task on day 1. Based on linear mixed model.

| Observational learning task D1 | F | Df | p | Post-hoc test of interaction (corrected) | Post-hoc test of interaction (corrected) |
| --- | --- | --- | --- | --- | --- |
| FEAR RATING |  |  |  | CS+ - CS-, pre | CS+ - CS-, post |
| Stimulus | 0.0227 | 1,267 | 0.8804 |  |  |
| Time | 1.1405 | 1,267 | 0.2865 |  |  |
| Stimulus*time | 24.0361 | 1,267 | <0.001 | t(267)=0.151,p=0.880 | t(267)=7.084,p<0.001 |
| DISCOMFORT RATING |  |  |  |  |  |
| Stimulus | 0.0552 | 1,267 | 0.8144 |  |  |
| Time | 18.4500 | 1,267 | <0.001 |  |  |
| Stimulus*time | 27.6412 | 1,267 | <0.001 | t(267)=-0.235,p=0.814 | t(267)=7.200,p<0.001 |
| PHYS. AROUSAL RATING |  |  |  |  |  |
| Stimulus | 0.3128 | 1,267 | 0.5764 |  |  |
| Time | 1.4327 | 1,267 | 0.2324 |  |  |
| Stimulus*time | 21.1139 | 1,267 | <0.001 | t(267)=0.559,p=0.576 | t(267)=7.058,p<0.001 |

Main effect of trial in steps analysis

We included CS door decision (2 levels: CS+ door/CS- door), the trial (20 levels: 1-20) and the pathlength (2 levels: long path/short path) into the model (steps~(1|subject) + CSdoor*pathlength+trial) and found a main effect of trial (F(19,1608.20)= 16.672, p<0.001) (Supplementary Figure S2).


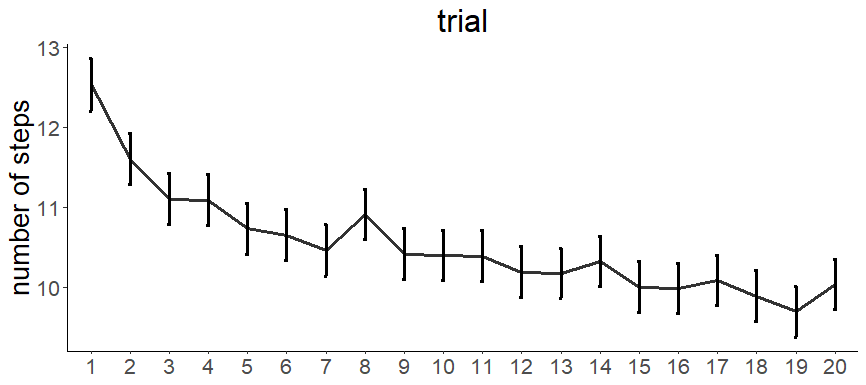


Supplementary Figure S2: Main effect of trial in the steps analysis shows that the number of steps is decreasing over time likely due to decrease in effort willing to take after several repetitions.

Supplementary Table S2: Results of ANOVA Type 3, based on the linear mixed model. Only effects with p<0.1 are shown. Dependent variable is number of steps (steps~(1|subject)+CSdoor*pathlength+trial).

|  | F | Df | p |
| --- | --- | --- | --- |
| CS door decision | 118.632 | 1,1635.06 | <0.001 |
| pathlength | 126.564 | 1,1614.40 | <0.001 |
| trial | 16.672 | 19,1608.20 | <0.001 |
| CS door decision * pathlength | 24.588 | 1,1618.77 | <0.001 |
| Post-hoc tests of the interaction: | |  |  |
| CS+,short – CS-,short | t(1635) = -10.892, p_corr_ < 0.001 | | |
| CS+,short – CS+,long | t(1614) = -11.250, p_corr_ < 0.001 | | |
| CS-,short – CS-,long | t(1614) = -4.057, p_corr_ = 0.001 | | |
| CS+,long – CS-,long | t(1634) = -4.650, p_corr_ < 0.001 | | |

Supplementary Table S3: Results of ANOVA Type 3, based on the linear mixed model. Only effects with p<0.1 are shown. Dependent variable is number of steps. Differential ratings are included in the model. Only results that included rating effects are shown.

Model: lmer(steps~(1|subject)+ratingdifference*CS*pathlength+trial)

|  | F | Df | p |
| --- | --- | --- | --- |
| Discomfort, post D2 |  | | |
| Rating*CS | 4.0176 | 1,1628.70 | 0.04519 |
| Rating*CS*pathlength | 3.9566 | 1,1615.89 | 0.04686 |
| Fear, post D2 |  | | |
| Rating*CS | 2.9737 | 1,1625.63 | 0.08482 |
| Rating*CS*pathlength | 3.0746 | 1,1614.08 | 0.07972 |
| Physical Arousal, pre D1 |  | | |
| Rating*CS*pathlength | 2.9676 | 1,1617.95 | 0.08514 |
| Physical Arousal, post D2 |  | | |
| Rating*CS | 4.3780 | 1,1630.88 | 0.03656 |
| Rating*CS*pathlength | 4.0594 | 1,1615.63 | 0.04409 |

Supplementary Table S4: Results of ANOVA Type 3, based on the linear mixed model. Only effects with p<0.1 are shown. Dependent variable is the reaction time per step before or after door decision (lmer(reactiontime~(1|subject)+Csdoor*pathlength+steps+trial).

| RT PRE DOOR DECISION | F | Df,Dfres | p |
| --- | --- | --- | --- |
| trial | 26.4817 | 1,8784.4 | <0.001 |
| steps | 33.8796 | 19,8733.3 | <0.001 |
| RT POST DOOR DECISION |  |  |  |
| trial | 168.1539 | 1,7237.9 | <0.001 |
| steps | 20.9536 | 19,7347.5 | <0.001 |

Supplementary Table S5: Results of ANOVA Type 3, based on the linear mixed model. Only effects with p<0.1 are shown. Dependent variable is the wallfactor, i.e. how close to the wall subjects moved. (lmer(wallfactor~(1|subject)+CS *pathlength+trial).

|  | F | Df,Dfres | p |
| --- | --- | --- | --- |
| CS | 70.1281 | 1, 1654.93 | <0.001 |
| Pathlength | 33.4875 | 1, 1623.36 | <0.001 |
| Block | 5.4230 | 19,1613.31 | <0.001 |
| CS*pathlength | 7.1402 | 1,1630.46 | 0.007612 |
